# Biomarkers of erosive arthritis in systemic lupus erythematosus: Application of machine learning models

**DOI:** 10.1371/journal.pone.0207926

**Published:** 2018-12-04

**Authors:** Fulvia Ceccarelli, Marco Sciandrone, Carlo Perricone, Giulio Galvan, Enrica Cipriano, Alessandro Galligari, Tommaso Levato, Tania Colasanti, Laura Massaro, Francesco Natalucci, Francesca Romana Spinelli, Cristiano Alessandri, Guido Valesini, Fabrizio Conti

**Affiliations:** 1 Lupus Clinic, Rheumatology, Dipartimento di Medicina Interna e Specialità Mediche, Sapienza Università di Roma, Rome, Italy; 2 Dipartimento di Ingegneria dell'Informazione, Università di Firenze, Florence, Italy; University College London, UNITED KINGDOM

## Abstract

**Objective:**

Limited evidences are available on biomarkers to recognize Systemic Lupus erythematosus (SLE) patients at risk to develop erosive arthritis. Anti-citrullinated peptide antibodies (ACPA) have been widely investigated and identified in up to 50% of X-ray detected erosive arthritis; conversely, few studies evaluated anti-carbamylated proteins antibodies (anti-CarP). Here, we considered the application of machine learning models to identify relevant factors in the development of ultrasonography (US)-detected erosive damage in a large cohort of SLE patients with joint involvement.

**Methods:**

We enrolled consecutive SLE patients with arthritis/arthralgia. All patients underwent joint (DAS28, STR) and laboratory assessment (detection of ACPA, anti-CarP, Rheumatoid Factor, SLE-related antibodies). The bone surfaces of metacarpophalangeal and proximal interphalangeal joints were assessed by US: the presence of erosions was registered with a dichotomous value (0/1), obtaining a total score (0–20). Concerning machine learning techniques, we applied and compared *Logistic Regression* and *Decision Trees* in conjunction with the feature selection *Forward Wrapper* method.

**Results:**

We enrolled 120 SLE patients [M/F 8/112, median age 47.0 years (IQR 15.0); median disease duration 120.0 months (IQR 156.0)], 73.3% of them referring at least one episode of arthritis. Erosive damage was identified in 25.8% of patients (mean±SD 0.7±1.6), all of them with clinically evident arthritis. We applied *Logistic Regression* in conjunction with the *Forward Wrapper* method, obtaining an AUC value of 0.806±0.02. As a result of the learning procedure, we evaluated the relevance of the different factors: this value was higher than 35% for ACPA and anti-CarP.

**Conclusion:**

The application of Machine Learning Models allowed to identify factors associated with US-detected erosive bone damage in a large SLE cohort and their relevance in determining this phenotype. Although the scope of this study is limited by the small sample size and its cross-sectional nature, the results suggest the relevance of ACPA and anti-CarP antibodies in the development of erosive damage as also pointed out in other studies.

## Introduction

Joint involvement is one of the most common features in patients affected by Systemic Lupus Erythematosus (SLE): a high proportion of patients (69–95%) could experience this manifestation during disease course. A great heterogeneity characterizes this manifestation, moving from arthralgia to more severe arthropathy, with possible development of erosive damage [1].

For a long time, the presence of an erosive arthritis in SLE patients has been considered a rare condition and generally identified in subjects overlapping with Rheumatoid Arthritis (RA). The introduction of more sensitive imaging techniques in the assessment of inflammatory arthritis, such as ultrasonography (US), allowed the identification of erosive damage in up to 40% of patients with SLE-related arthritis [2].

Nevertheless, few data are available concerning specific biomarkers able to recognize patients at risk to develop erosive damage. Several studies investigated the role of RA specific autoantibodies, moving from their relevance in the identification of individuals at risk to develop RA and in determining erosive arthritis [3].

The presence of anti-citrullinated peptide antibodies (ACPA) has been analyzed in SLE patients, identifying this biomarker in up to 50% of SLE patients with X-ray detected erosive arthritis [1,4,5]. Conversely, few data are available concerning the association between anti-carbamylated proteins antibodies (anti-CarP) and bone erosions: Ziegelasch and colleagues have recently identified a significant association between X-ray detected erosive damage and anti-CarP in a small SLE cohort [6]. More recently, we confirmed this association in a large SLE population with joint involvement in which the damage was assessed by US [7].

Machine learning methodologies have already been applied in the medical setting. Artificial Neural Networks (ANNs) have been used in SLE cohorts to predict specific outcomes, such as chronic damage development or 3 years kidney graft survival in recipients affected by SLE [8,9].

Moreover, these mathematical models can be used to select the factors able to identify the presence of a specific outcome and to rate the relevance or ranking of different factors in determining it. Similar approaches have also been exploited in specific medical conditions, such as gene selection task in DNA microarray datasets, selection of genes associated with diffuse large B-cell lymphoma, and, finally, in the analysis of Alzheimer’s disease progression [10–12].

Moving from these premises, we considered the application of machine learning models to identify relevant factors in the development of US-detected erosive damage in a large single center cohort of 120 SLE patients with joint involvement. In this study, we employed Logistic Regression and Decision Trees, both machine learning models for classification which are easily interpretable, in conjunction with an iterative feature selection technique, in order to recognize factors associated with erosive bone damage.

## Materials and methods

Consecutive SLE patients with a clinical history of joint involvement, attending at the Lupus Clinic of the Rheumatology Unit, Sapienza University of Rome (*Sapienza Lupus Cohort*) were enrolled in the present study. SLE diagnosis was performed according to the revised 1997 American College of Rheumatology (ACR) criteria [13].

The study was performed according to the protocol and good clinical practice principles and Declaration of Helsinki statements and was approved by the Ethic committee of the Sapienza University of Rome, Policlinico Umberto I, Rome, Italy. All the patients signed an informed consent.

The clinical and laboratory data of enrolled patients were collected in a standardized computerized electronically filled form, including demographics, past medical history with the date of diagnosis, co-morbidities, previous and concomitant treatments, serological status [C3/C4 levels (radial immunodiffusion), ANA (IIF on HEp-2), anti-dsDNA (IIF on *Crithidia Luciliae*), anti-Ro/SSA, anti-La/SSB, anti-Sm, and anti-RNP, anti-Cardiolipin (anti-CL) and anti-β2 Glycoprotein-I (anti-β2GPI) (ELISA assay), lupus anticoagulant (LA) according to the guidelines of the International Society on Thrombosis and Hemostasis].

Patients were divided according to the presence of arthralgia and arthritis. Arthralgia was defined as the presence of recurrent (minimum three episodes) or persistent (minimum 6 weeks) pain or stiffness (lasting at least 30 minutes) of at least one joint during patient’s clinical history; arthritis as the occurrence of at least 1 episode of clinical synovitis (swelling, effusion or tenderness) and at least 30 minutes of morning stiffness of at least 1 joint.

The activity of joint involvement was assessed by using the disease activity score on 28 joints (DAS28) and the swollen to tender ratio (STR), both previously applied in SLE cohorts with joint involvement [14,15].

SLE Disease Activity Index 2000 (SLEDAI-2k) was used to assess disease activity, while chronic damage was evaluated by SLICC Damage Index (SDI) [16,17].

Each subject underwent peripheral blood sample collection. Rheumatoid Factor (RF) and ACPA were detected by using commercial ELISA kits (Diamedix, Miami, USA; DELTA BIOLOGICALS, Rome, Italy, respectively): the results were evaluated according to the manufacturers’ instructions. For ACPA, values above 25 U/mL were considered positive, while for RF, values above 10 U/mL.

Anti-CarP antibodies were detected by a home-made ELISA using carbamylated foetal calf serum (Ca-FCS) and non-modified FCS as antigens. Ca-FCS was obtained using the method described by Shi et al [18]. A titration curve of two positive reference sera with medium–high ELISA immunoreactivity for Ca-FCS was performed to show the performance of the tests and to transform the absorbance of Ca-FCS to arbitrary units per milliliter (aU/mL). The cut-off was established as the mean OD + 3 standard deviations (SD) of fifty-six age- and sex-matched healthy subjects (blood donors) and then the obtained value was converted into aU/mL (corresponding to 340 aU/mL).

US imaging was performed in all SLE patients by using a MyLab70 XVG machine (Esaote S.p.A., Florence, Italy) equipped with a 6–18 MHz multifrequency linear array transducer. By using a fixed 18-MHz frequency, bone surfaces of metacarpophalangeal (MCP) and proximal interphalangeal (PIP) were studied on multiplanar scans, according with the EULAR US guidelines [19]. Each joint was scanned in both the longitudinal and transverse planes from the medial to lateral sides on both volar and dorsal aspects to enable maximum coverage of the joint surface area. At each joint, according with OMERACT definition, the presence of erosions was registered with a dichotomous value (0/1), allowing the possibility to obtain a total score, ranging from 0 to 20 [20].

### Statistical analysis

The statistical analyses were performed using the version 5.0 of the GraphPad statistical package (La Jolla, California). Normally distributed variables were summarized using the mean ± SD, and non-normally distributed variables by the median and interquartile range (IQR). Frequencies were expressed by percentage.

### Machine learning

In order to understand factors leading to the development of erosive damage in SLE patients, we employed machine learning techniques. In particular we used binary classification models, which can be applied to learn a function that partitions of the data in two groups: in our case the two groups were SLE patients with and without bone erosions. The separating function depends on the features describing the data. Once a function has been identified, we can deepen our understanding on the factors more influencing the function behavior and on the modality by which the function relates to the different features.

One question naturally arises: what is the degree of trust we have in the so identified factors? Of course, the relevance of the factors which are extracted by looking at the separating function is only good as the function itself. For this reason, a model has to be evaluated on a test set, as it is usually done in machine learning. Better models will lead to more reliable identified factors and the test accuracy can be used to measure the goodness of the obtained ranking.

In light of the possible influence in the performance of machine learning models due to the presence of irrelevant features in the data, especially when few cases (patients) are available, a feature selection technique was employed, in order to select among all the available features a smaller subset of meaningful ones.

After the application of feature selection, the model tailored on the selected subset of features can be applied to gain insights on the relative importance of each selected feature. In the following, we describe the *Logistic Regression* and *Decision Tree* models for classification, and the *Forward Wrapper* feature selection technique. We choose these two models for two reasons: 1) their natural interpretability, a desirable characteristic when the goal is to assess the importance of each feature in the outcome produced by the model and 2) more complex models, such as neural networks, usually have poor performances when the sample size is small.

#### Classification

A classification model is trained with a dataset of m examples[(x^i^, y^i^, I = 1, …, m], where x^i^ contains the variables (called features) of patient i and y^i^ is a binary variable which indicates whether such patient has developed erosion or not. Once a model is trained, we can evaluate its generalization capabilities by testing it on unseen data.

#### Logistic Regression

*Logistic Regression* is a linear classifier which aims to find a function *h*_*w*,*b*_ such that

$$h_{w,b}(x^{i})\approx y^{i}\forall i\;=\;1,\;.\;.\;.,m.$$

In particular, the function *h*_*w*,*b*_(*x*) takes the following form:

$$h_{w,b}(x)=\frac{1}{1+e^{-(w^{T}x+b)}}$$

where the weight vector *w* and bias *b* are learned parameters, tuned with an iterative procedure. To avoid over-fitting the training data, the model also employs a penalty term *l*(*w*), to control the complexity of the model. Here we use *l*(*w*) = *λ*||*w*||^2^, where *λ* > 0 is an hyper-parameter which has to be tuned during model selection. Such procedure is performed by evaluating different configurations of hyper-parameters on a held-out portion of the train set (or several different portions, as in “leave-one-out” or k-fold). Here we select the hyper-parameter configurations among a grid of candidates (grid-search).

After the training phase, the value *h*_*w*,*b*_(*x*) can be seen as a probability estimate on the class of the input vector *x*: if *h*_*w*,*b*_(*x*) ≥ 0.5; we classify *x* as positive; otherwise, we classify it as negative.

The weight vector *w* of the fitted *Logistic Regression* model can be used to measure the “importance” of each feature. Several approaches to determine the relative importance of the explanatory variables from the coefficients of a logistic regression model exists in the literature. Here we follow the method proposed in [21].

#### Decision Trees

A *Decision Tree* is a non-parametric machine learning model. It is structured as a tree where each internal node, including the root, is a decision test over one of the features. In particular, for continuous features, the test is of the form x_i_ ≤ alpha for some value alpha For categorical features, instead, the test is of the form x_i = C,_ where *c* is one of the possible categories. The training phase is responsible for choosing the right decision tests, i.e., the feature that define a node and its associated “threshold”. After the tree is constructed, starting from the root, an example *x* is recursively assigned to a subtree, according to the the different decision tests that are applied at each visited node. In the end,x will be assigned to one of the terminal nodes of the tree, also called “leaves”. Each leaf has an associated response y, chosen with a majority vote over the values y^i^ corresponding to the training examples x^i^ that end up in the leaf in question. A number of different regularization techniques can be used. For example, we can limit the maximum depth of the tree, and the minimum number of training examples that define a leaf. These techniques help in reducing over-fitting, which is of real concern given the fact that decision trees are non-parametric. The maximum depth and the minimum number of examples in a leaf are hyper-parameters that, as in the logistic regression case, must be tuned during model selection. *Decision Trees* are also naturally interpretable. The features appear in a hierarchical fashion in the tree, where the features closer to the root can be seen as more important than the ones appearing in the lower part of the tree. Moreover, a tree implicitly performs feature selection: some features may not be present in any of the nodes of the tree.

#### Feature selection

Starting from the entire set of features, the feature selection is performed with the aim of selecting a subset of relevant features. The main reasons concern the simplification of the resulting model, the individuation of the most important features and the achievement of a higher generalization capability of the machine learning models.

Since an extensive search among all 2^n^ possible subsets of features, where *n* is the total number of features, is generally impracticable, several methods have been proposed in the machine learning literature in order to overcome this limitation. These methods can be divided into three main classes [22]:

- **Filter Methods**: the selection of the features does not rely on the use of a model and generally a score is assigned to each feature in order to obtain a ranking.
- **Wrapper Methods**: several subsets of features are evaluated and compared by assigning a score based on the accuracy achieved with a predictive model; among methodical, stochastic and heuristic search processes of the subsets; an example is the forward (or backward) method where features are iteratively added (or removed) based on the obtained model accuracy.
- **Embedded Methods:** this class of methods tries to combine the advantages of both previous methods, in which feature selection is embedded in the learning process.

The *Forward Wrapper* belongs to the class of wrapper methods. It starts with an empty set of features. Then, at each iteration, all the features not included in the current feature set are added independently and the one which leads to the best performance of the employed predictive model is inserted.

The process is not stopped in case the insertion of a new feature leads to a worse performance score. Thus, the process requires an overall number of steps equal to the total number of features. At the end, the overall best score achieved through iterations is considered and the correspondent subset of features is chosen as the result of the feature selection.

## Results

We enrolled 120 SLE patients with joint involvement [M/F 8/112, median age 47.0 years (IQR 15.0); median disease duration 120.0 months (IQR 177.0)]. Eighty-eight patients (73.3%) referred at least one episode of arthritis during disease history. The main clinical, laboratory and therapeutic features of the whole cohort were described in Table 1.

**Table 1:** Demographic features, clinical and laboratory manifestations and treatment of 120 SLE patients.

**Clinical manifestations—N (%)**	
Skin involvement	88 (73.3)
Serositis	21 (17.5)
Hematological manifestations	72 (60.0)
Neuropsychiatric involvement	11 (9.2)
Renal involvement	28 (23.3)
**Laboratory manifestations—N (%)**	
Anti-DNA	86 (71.7)
Anti-Sm	19 (15.8)
Anti-SSA	43 (35.8)
Anti-SSB	26 (21.7)
Anti-RNP	17 (14.2)
Anti-Cardiolipin IgG/IgM	44 (36.7)
Anti-β_2_ Glycoprotein I IgG/IgM	30 (25.0)
Lupus Anticoagulant	34 (28.3)
Low C3/C4 levels	66 (55.0)
**Treatments—N (%)**	
Glucocorticoids	108 (90.0)
Hydroxychloroquine	112 (93.3)
Cyclosporine A	28 (23.3)
Methotrexate	41 (34.2)
Cyclophosphamide	6 (5.0)
Mycophenolate Mofetil	31 (25.8)
Azathioprine	26 (21.7)
Rituximab	5 (4.2)
Belimumab	5 (4.2)

At the time of the study enrollment, a median SLEDAI-2k of 2.0 (IQR 4.0) was registered. Concerning the joint involvement activity, the whole population showed a median DAS28 of 3.42 (IQR 2.2) and a median STR of 0.08 (IQR 0.68). By using US assessment, an erosive damage was identified in 31 SLE patients (25.8%) with a mean ±SD of 0.7±1.6 (range 1–9). All these patients referred at least one episode of clinically evident arthritis.

Moving to the application of machine learning for the characterization of the erosive damage in SLE patients, we evaluated the generalization capabilities of the *Logistic Regression* and *Decision Trees* using 100 Monte Carlo repeated trials. Namely, we partition the data in 80% train and 20% test, preserving the positive-negative ratio, train the model using the training set and evaluate such model on the test set. We used two different metrics to evaluate the models, the area under the ROC curve (AUC) and the Matthews Correlation Coefficient (MCC), defined as

$$MCC\;=\;\frac{TPxTN-FPxFN}{\sqrt[{2}]{\left(TP+FP)(TP+FN)(TN+FP)(TN+FN\right)}},$$

where TP, TN, FP, FN are true positives, true negatives, false positives and false negatives, respectively. Both metrics are suitable for binary classification when the number of positives and negatives are of different sizes. AUC takes values in [0, 1] while MCC in [-1, 1]. Specifically, random predictions yield an AUC and MCC equal to 0.5 and 0, respectively, while for prefect predictions we have both metrics equal to 1.

The process is repeated 100 times collecting, at each trial, AUC and MCC values. At the end, we extract mean and confidence intervals for both metrics.

To choose the hyper-parameters of the model (at each trial) we employed, instead, a “leave-one-out” procedure since the number of examples that are available for training after the initial split is limited. Such procedure is, in fact, often employed when the number of available data is limited, thus, making it impracticable to separate a sufficiently large train-set to build robust models and a validation-set for evaluating their performances. “leave-one-out” procedure overcomes these problems excluding a single example from the train-set, training the model on the slightly reduced dataset and computing the prediction of the model on the example that was excluded.

In the first experiment, we employed the implementations of *Logistic Regression* and *Decision Tree* available in the 0.18 version of the *scikit-learn* library, using all the available features. The results are reported in Table 2.

**Table 2:** AUC and MCC values (mean ± CI) obtained by *Logistic Regression* and *Decision Tree*.

Model	AUC [0, 1]	MCC [-1, 1]
Logistic Regression	0.728±0.02	0.257±0.04
Decision Tree	0.719±0.02	0.274±0.04

Next, in order to improve the generalization capability, we used the *Forward Wrapper* method with both *Logistic Regression* and *Decision Trees*. We used MCC as the metric to evaluate each subset of features at each step of the algorithm. Table 3 reports the numerical results for the subset of features selected by *Forward Wrapper*.

**Table 3:** Comparison between *Logistic Regression* and *Decision Trees* with the *Forward Wrapper* method.

Model	Feature selection	AUC [0, 1]	MCC [-1, 1]
Logistic Regression	Forward Wrapper	0.806±0.02	0.481±0.03
Decision Tree	Forward Wrapper	0.752±0.02	0.301±0.04

We note that the employment of the feature selection approach resulted in an improvement of the generalization capability both in terms of AUC and MCC for both models, although *Logistic Regression* performs better w.r.t. both metrics.

The mean MCC score for each step of the feature selection process, for *Logistic Regression*, is reported in Supplementary material (S1 Fig). The subset of features selected by the algorithm includes anti-CarP, ACPA, arthralgia, Jaccoud’s arthropathy, anti-Sm, and neurological manifestations.

**Fig 1:**
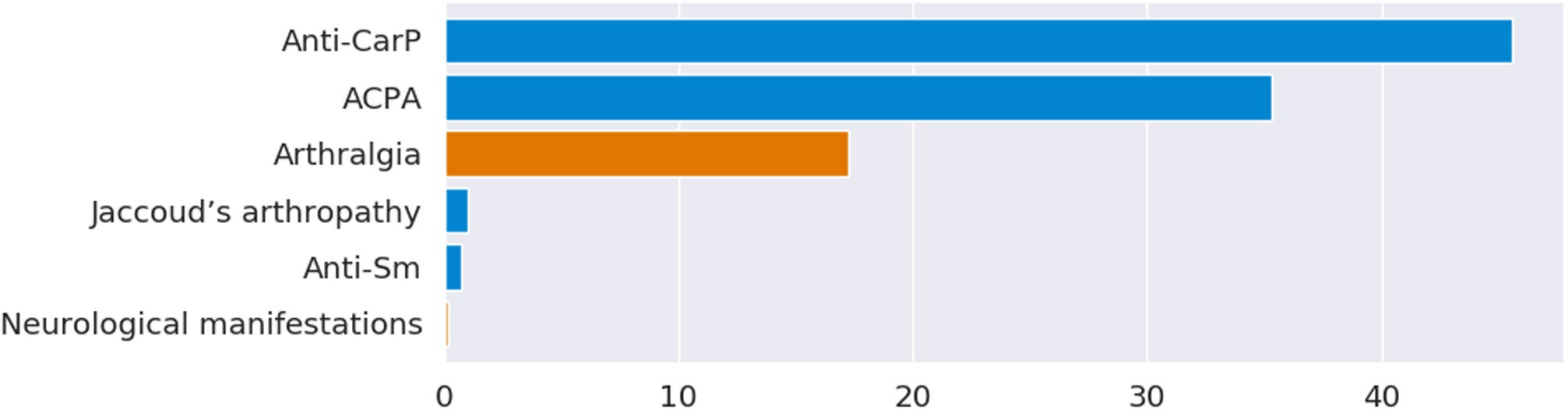
Relative importance (%) of the selected features. Blue colored ones are positively associated with the development of bone erosions while orange ones are negatively associated.

Since the obtained results in terms of generalization capability are satisfactory, the *Logistic Regression* model combined with the feature selection is able to characterize well the relation between the erosive damage and the selected features. Thus, such model can be confidently used to evaluate the relevance of each feature in the development of erosive damage in SLE patients, which is the primary aim of the analysis. To do this, we considered the features selected by the *Forward Wrapper* and *Logistic Regression* and we trained a new model on all the available data obtaining a weight vector. The hyper-parameters for this final model have been chosen with a leave-one-out scheme performed on all the available examples. The relative importance of the selected feature is computed analyzing such weight vector. Fig 1 reports the relative importance (%) of the selected features. Note that the coefficients of the model can be further used to determine if a feature is positively (blue colored in Fig 1) associated with the development of bone erosion or negatively associated (orange colored).

As reported in Fig 1, anti-CarP, ACPA and arthralgia resulted the most relevant features for our model. We note that while a patient with high levels of anti-CarP and ACPA will be likely classified as positive by the model, arthralgia, instead, has an inverse effect, confirming the observation that only SLE patients with at least one arthritis episode develop erosive damage.

The combination of the 6 features reported in Fig 1 (anti-CarP, ACPA, Jaccoud’s arthropathy, anti-Sm, arthralgia, neurological manifestations) identified an AUC value of 0.806±0.02 and a MCC value of 0.481±0.03. To compare our results whit those obtained by Verheul and colleagues, we also performed a test with the three antibodies anti-CarP, ACPA and RF [3]. This test showed a decrease in AUC (0.676±0.02) and MCC value (0.22±0.05).

To summarize, the overall process applied in the present study consisted in

1. selecting the best subset of features with the *Forward Wrapper* coupled with *Logistic Regression* and *Decision Tree*;
2. fitting the best model on all the available data;
3. use such model to assess the relative importance of the selected feature.

**Fig 2:**
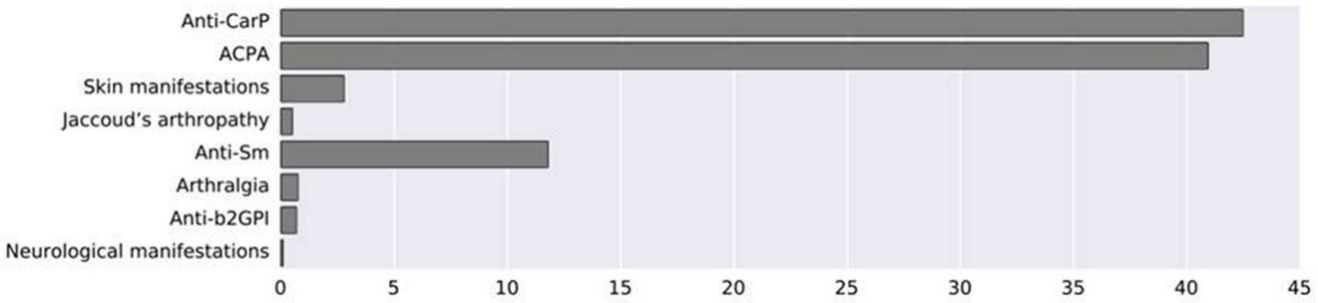
Complete pipeline of the method.

The complete process is sketched in Fig 2.

## Discussion

To the best of our knowledge, this is the first study aimed at applying the *Machine Learning* models in order to identify factors associated with US-detected erosive bone damage in a large SLE cohort and their relevance in determining this specific phenotype.

In particular, decision trees model was compared with logistic regression, in conjunction with forward wrapper feature selection.

Thanks to this approach, we confirm the relevance of ACPA and anti-CarP in determining SLE-related erosive damage, suggesting their pathogenic role in the development of this phenotype. In particular, these autoantibodies showed a positive association and a similar relative importance, which was higher than 40% for both autoantibodies.

These results reinforce the role of these autoantibodies as biomarkers of bone damage, suggesting a pathological link between their presence and bone erosions development.

Despite the high frequency of SLE-related joint involvement, data concerning pathogenic mechanisms and specific biomarkers are lacking. In the last years, moving from evidences deriving from RA, the role of post-translational modifications has been suggested: in particular, citrullination and carbamylation could be able to induce neo-antigens generation and autoantibodies production in genetically susceptible individuals [23].

Indeed, ACPA have been frequently observed in SLE patients with X-ray detected erosive arthritis [1,2,4,5]. Nonetheless, a relevant percentage of SLE patients with erosive arthritis is ACPA negative, suggesting a different pathogenic *scenario*. In order to fill this space, also anti-CarP have been evaluated in SLE cohorts. In a previous study, we found the presence of anti-CarP in 46.1% of SLE patients with joint involvement, a prevalence similar to that identified in RA patients and significantly higher respect to healthy controls [24]. Moreover, Ziegelasch and colleagues identified an association between anti-CarP and radiographically detected erosions in a small cohort of SLE patients [6].

By using machine learning techniques, in particular the *Forward Wrapper* method, we confirmed this association, and, interestingly, we estimated the weight of ACPA and anti-CarP in determining a more aggressive phenotype in SLE-related joint involvement. These results reinforce the need to better understand the pathogenic mechanisms that could explain this association. We could hypothesize that ACPA and anti-CarP exert an action on osteoclasts, leading to erosive damage development.

Furthermore, a mild relevance was identified for the presence of Jaccoud’s arthropathy in erosive damage development. This result is in agreement with our previous study specifically evaluating SLE patients with this arthropathy. We found US-detected erosive damage in almost 60% of patients evaluated, observed prevalently at level of first and second MCP joint; moreover, the erosive damage was significantly associated with ACPA [25]. The low relative importance obtained for this feature could be related to its low frequency, ranging from 2 to 35% in described SLE cohorts [25].

On the other hand, if a patient did not present arthritis but only arthralgia during the clinical history, it is unlikely that bone erosions will develop. This is in agreement with previous studies performed on cohorts of patients with inflammatory arthritis and other diseases such as inflammatory bowel disease, in which erosions could not be detected by MRI [26].

Of note, in the present study we evaluated the erosive damage by using US assessment: this imaging technique demonstrated a higher sensitive in comparison of radiographic assessment in the evaluation of bone erosions at level of MCP and PIP in RA patients, especially during early disease phase [27].

A limit of this study is the cross-sectional design. The realization of a longitudinal analysis, in fact, is certainly needed in order to further understand the importance of the different prognostic factors of SLE-related erosive damage. Moreover the relatively small sample size discourages the use of more complex machine learning models that, when trained with large datasets, could arguably perform better than the simpler methods we consider here. Finally a larger sample size could surely lead to more robust results.

In conclusion, despite the small sample size and the cross-sectional design of this study, the application of machine learning models provides a new point of view in the research of biomarkers for SLE-related erosive arthritis, confirming the possible role of ACPA and anti-CarP on this specific phenotype.

## Supporting information

S1 Fig Mean MCC score for each step of the feature selection process for Logistic Regression. (TIFF)

S1 Data Source Demographic features, clinical and laboratory manifestations and treatment of enrolled patients. (XLSX)

## References

[1] Ceccarelli F, Perricone C, Cipriano E, Massaro L, Natalucci F, Capalbo G, et al Joint involvement in systemic lupus erythematosus: From pathogenesis to clinical assessment. Semin Arthritis Rheum 2017;47:53–64. 10.1016/j.semarthrit.2017.03.022 28465078 10.1016/j.semarthrit.2017.03.022

[2] Wright S, Filippucci E, Grassi W, Grey A, Bell A. Hand arthritis in systemic lupus erythematosus: an ultrasound pictorial essay. Lupus 2006; 15:501–6. 10.1191/0961203306lu2340oa 16942002 10.1191/0961203306lu2340oa

[3] Verheul MK, Böhringer S, van Delft MAM, Jones JD, Rigby WFC, Gan RW, et al The combination of three autoantibodies, ACPA, RF and anti-CarP antibodies is highly specific for rheumatoid arthritis: implications for very early identification of individuals at risk to develop rheumatoid arthritis. Arthritis Rheumatol. 2018 5 21. 10.1002/art.40562 29781231

[4] Taraborelli M, Inverardi F, Fredi M, Ceribelli A, Cavazzana I, Tincani A, et al Anti-cyclic citrullinated peptide antibodies in systemic lupus erythematosus patients with articular involvement: a predictive marker for erosive disease? Reumatismo 2012; 64:321–5. 10.4081/reumatismo.2012.321 23256108 10.4081/reumatismo.2012.321

[5] Amezcua-Guerra LM, Springall R, Marquez-Velasco R, Gomez-Garcia L, Vargas A, Bojalil R. Presence of antibodies against cyclic citrullinated peptides in patients with 'rhupus': across-sectional study. Arthritis Res Ther 2006; 8:R144 10.1186/ar2036 16934155 10.1186/ar2036 PMC1779435

[6] Ziegelasch M, van Delft MA, Wallin P, Skogh T, Magro-Checa C, Steup-Beekman GM, et al Antibodies against carbamylated proteins and cyclic citrullinated peptides in systemic lupus erythematosus: results from two well-defined European cohorts. Arthritis Res Ther. 2016;18:289 10.1186/s13075-016-1192-x 27912793 10.1186/s13075-016-1192-x PMC5135817

[7] Ceccarelli F, Perricone C, Colasanti T, Massaro L, Cipriano E, Pendolino M, et al Anti-carbamylated protein antibodies as a new biomarker of erosive joint damage in systemic lupus erythematosus. Arthritis Res Ther2018;20:126. 10.1186/s13075-018-1622-z PMC6001021 29898764

[8] Ceccarelli F, Sciandrone M, Perricone C, Galvan G, Morelli F, Vicente LN, et al Prediction of chronic damage in systemic lupus erythematosus by using machine-learning models. PLoS One. 2017; 12:e0174200 10.1371/journal.pone.0174200 28329014 10.1371/journal.pone.0174200 PMC5362169

[9] Tang H, Poynton MR, Hurdle JF, Baird BC, Koford JK, Goldfarb-Rumyantzev AS. Predicting three-year kidney graft survival in recipients with systemic lupus erythematosus. ASAIO J 2011; 57:300–9. 10.1097/MAT.0b013e318222db30 21701272 10.1097/MAT.0b013e318222db30

[10] Blazadonakis ME, Zervakis M. Wrapper filtering criteria via linear neuron and kernel approaches. Comput Biol Med. 2008; 38:894–912. 10.1016/j.compbiomed.2008.05.005 18656182 10.1016/j.compbiomed.2008.05.005

[11] Shipp MA, Ross KN, Tamayo P, Weng AP, Kutok JL, Aguiar RC, et al Diffuse large B-cell lymphoma outcome prediction by gene-expression profiling and supervised machine learning. Nat Med 2002; 8:68–74. 10.1038/nm0102-68 11786909 10.1038/nm0102-68

[12] Jin Y, Su Y, Zhou XH, Huang S. Alzheimer’s Disease Neuroimaging Initiative. Heterogeneous multimodal biomarkers analysis for Alzheimer's disease via Bayesian network. EURASIP J Bioinform Syst Biol. 2016;2016:12 27610127 10.1186/s13637-016-0046-9 PMC4992017

[13] Hochberg MC. Updating the American College of Rheumatology revised criteria for the classification of systemic lupus erythematosus. Arthritis Rheum 1997; 40:1725. 10.1002/art.1780400928 9324032

[14] Ceccarelli F, Perricone C, Massaro L, Pacucci VA, Cipriano E, Truglia S, et al The role of disease activity score 28 in the evaluation of articular involvement in systemic lupus erythematosus. ScientificWorldJournal 2014;2014:236842. 10.1155/2014/236842 PMC4235977 25530992

[15] Cipriano E, Ceccarelli F, Massaro L, Spinelli FR, Alessandri C, Perricone C, et al Joint involvement in patients affected by systemic lupus erythematosus: application of the swollen to tender joint count ratio. Reumatismo 2015; 67:62–7. 10.4081/reumatismo.2015.828 26492964 10.4081/reumatismo.2015.828

[16] Gladman DD, Ibanez D, Urowitz MB. Systemic lupus erythematosus disease activity index 2000. J Rheumatol 2002; 29:288–91. 11838846

[17] Gladman D, Ginzler E, Goldsmith C, Fortin P, Liang M, Urowitz M, et al The development and initial validation of the Systemic Lupus International Collaborating Clinics/American College of Rheumatology damage index for systemic lupus erythematosus. Arthritis and rheumatism. 1996;39(3):363–9. 8607884 10.1002/art.1780390303

[18] Shi J, Knevel R, Suwannalai P, et al Autoantibodies recognizing carbamylated proteins are present in sera of patients with rheumatoid arthritis and predict joint damage. Proc Natl Acad Sci USA 2011; 108: 17372–17377. 10.1073/pnas.1114465108 21987802 10.1073/pnas.1114465108 PMC3198314

[19] Backhaus M, Burmester GR, Gerber T, Grassi W, Machold KP, Swen WA, et al Guidelines for musculoskeletal ultrasound in rheumatology. Ann Rheum Dis 2001; 60: 641–649. 10.1136/ard.60.7.641 11406516 10.1136/ard.60.7.641 PMC1753749

[20] Wakefield RJ, Balint PV, Szkudlarek M, Filippucci E, Backhaus M, D'Agostino MA, et al Musculoskeletal ultrasound including definitions for ultrasonographic pathology. J Rheumatol 2005; 32: 2485–2487. 16331793

[21] Tonidandel S, LeBreton JM. Determining the Relative Importance of Predictors in Logistic Regression: An Extension of Relative Weight Analysis. Organizational Research Methods 2010;13:767–781.

[22] Guyon Isabelle, Elisseeff André. An introduction to variable and feature selection. J. Mach. Learn. Res. 3 (3 2003), 1157–1182.

[23] Mastrangelo A, Colasanti T, Barbati C, Pecani A, Sabatinelli D, Pendolino M, et al The Role of Posttranslational Protein Modifications in Rheumatological Diseases: Focus on Rheumatoid Arthritis. J Immunol Res. 2015;2015:712490 10.1155/2015/712490 26090496 10.1155/2015/712490 PMC4451265

[24] Massaro L, Ceccarelli F, Colasanti T, Pendolino M, Perricone C, Cipriano E, et al Anti-carbamylated protein antibodies in systemic lupus erythematosus patients with articular involvement. Lupus. 2018;27:105–111. 10.1177/0961203317713141 28592200 10.1177/0961203317713141

[25] Ceccarelli F, Massaro L, Perricone C, Pendolino M, Cipriano E, Truglia S, et al Jaccoud's arthropathy in systemic lupus erythematosus: clinical, laboratory and ultrasonographic features. Clin Exp Rheumatol 2017; 35:674–677. 28339366

[26] Brakenhoff LK, Stomp W, van Gaalen FA, Hommes DW, Bloem JL, van der Heijde DM, et al Magnetic resonance imaging of the hand joints in patients with inflammatory bowel disease and arthralgia: a pilot study. Scand J Rheumatol. 2014; 43:416–8. 10.3109/03009742.2014.882407 24720480 10.3109/03009742.2014.882407

[27] Backhaus M, Kamradt T, Sandrock D, Loreck D, Fritz J, Wolf KJ, et al Arthritis of the finger joints: a comprehensive approach comparing conventional radiography, scintigraphy, ultrasound, and contrast-enhanced magnetic resonance imaging. Arthritis Rheum. 1999;42:1232–45. 10.1002/1529-0131(199906)42:6<1232::AID-ANR21>3.0.CO;2-3 10366117 10.1002/1529-0131(199906)42:6<1232::AID-ANR21>3.0.CO;2-3

