# Correction: Biomarkers of erosive arthritis in systemic lupus erythematosus: Application of machine learning models

**DOI:** 10.1371/journal.pone.0211791

**Published:** 2019-01-30

**Authors:** Fulvia Ceccarelli, Marco Sciandrone, Carlo Perricone, Giulio Galvan, Enrica Cipriano, Alessandro Galligari, Tommaso Levato, Tania Colasanti, Laura Massaro, Francesco Natalucci, Francesca Romana Spinelli, Cristiano Alessandri, Guido Valesini, Fabrizio Conti

Fig 2 is incorrect. Please see the correct Fig 2 here.

**Fig 2:**
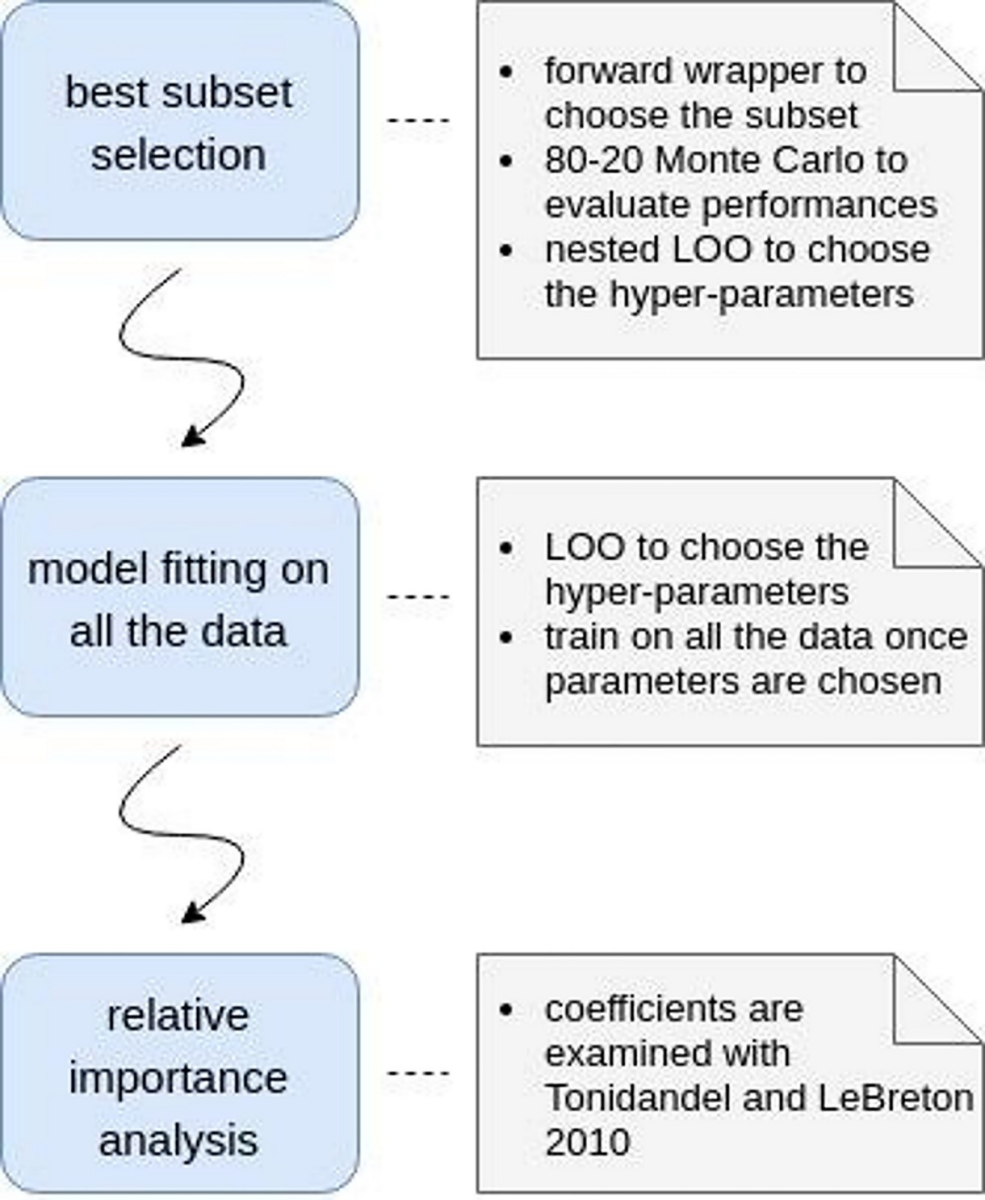
Complete pipeline of the method.
