# Supplementary figures and images for: Biomarkers of erosive arthritis in systemic lupus erythematosus: Application of machine learning models
Source: PLoS One. 2018 Dec 4;13(12):e0207926. doi: 10.1371/journal.pone.0207926 (PMC6279013; doi:10.1371/journal.pone.0207926)

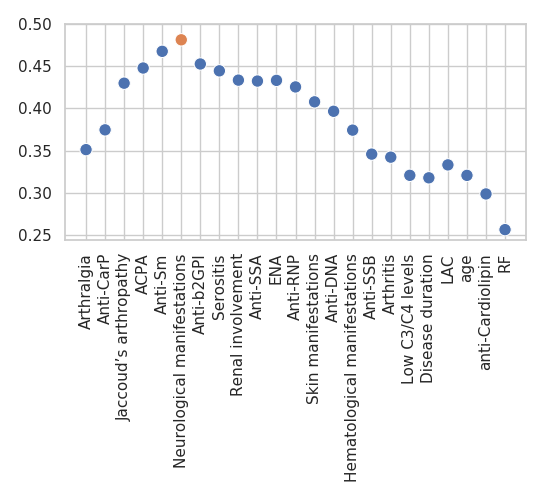

Supplement: S1 Fig — (TIFF) [file pone.0207926.s001.tiff]
